# Localized scleroderma in a cohort of juvenile idiopathic arthritis children

**DOI:** 10.1186/1546-0096-12-S1-P305

**Published:** 2014-09-17

**Authors:** Teresa Giani, Valeria Paganelli, Edoardo Marrani, Simonini Gabriele, Ilaria Pagnini, Achille Marino, Rolando Cimaz

**Affiliations:** 1 Pediatrics, Azienda Ospedaliera Meyer, Florence, Italy

## Introduction

Juvenile idiopathic arthritis (JIA) is the most common chronic rheumatic disease in childhood. Localized scleroderma also known as morphea is an inflammatory, fibrosing skin disorder that leads to sclerosis of the dermis and eventually of the underlying tissues. The prevalence is estimated at around 1-9/100,000 with the onset before 10 years of age in 2% of patients.The association between different autoimmune diseases is well described, but few studies have been performed to investigate the relationship between JIA and other autoimmune diseases, in particular very little is written about the possible association between arthritis and morphea. In these cases articular involvement in terms of arthralgia, impaired joint mobility, or joint contracture, may be related to a mechanical etiology secondary to the contracture of the overlying skin.

## Objectives

To describe the association of morphea and JIA in our cohort.

## Methods

We analyzed a total of 440 patients carrying the diagnosis of JIA followed in our Centre from January 1, 2010 to January 1, 2014.

## Results

We found four children that were diagnosed to have also morphea: a 12-year-old boy with polyarticular JIA that presented on the inner surface of the right thigh a well demarcated plaque of about 5 cm in diameter, a 8-year- old girl with polyarticular JIA who had a linear lesion extending from the postero-lateral surface of the right elbow to the back of the wrist as well as a small patch of 2 cm in diameter on the volar surface of the same elbow, a 7-year-old female with oligoarticular JIA with a patch on the anterior surface of the right leg and two similar, smaller lesions over the dorsum of the left foot and one over the dorsum of the right foot, and an 11-year-old chinese boy with HLA-B27 positive enthesitis-related arthritis that presented a linear skin lesion on the medial surface of the left knee. Except for the boy with the polyarticular form in which morphea preceded by one year the joint involvement, in the other cases morphea followed the arthritis, appearing in periods in which the disease was in remission and off any therapy.The boy with polyarticular arthritis was not treated because the lesion appeared already in the atrophic stage at the time of the first visit, while the other 3 children were placed on therapy with methotrexate at a dose of 15 mg/sq per week subcutaneously for approximately one year in combination with 3 months of oral prednisone, 1 mg/kg/day. All of them achieved a clinical remission for a mean duration of 12 months.

## Conclusion

In our series of children with JIA we documented the appearance of morphea in 0.9% of cases, which is more than would be expected by chance alone, and we observed an association with different types of articular onset. In these patients arthritis was localized in different and distant sites from those affected by morphea, removing the possibility of a mechanical phenomenon or of a misdiagnosis. Although localized scleroderma is considered an autoimmune disease limited to the skin the association with JIA could suggest a systemic imbalance of the immune system.

## Disclosure of interest

None declared.

